# Influence of channel height on mixing efficiency and synthesis of iron oxide nanoparticles using droplet-based microfluidics†

**DOI:** 10.1039/d0ra02470h

**Published:** 2020-04-17

**Authors:** O. Kašpar, A. H. Koyuncu, A. Hubatová-Vacková, M. Balouch, V. Tokárová

**Affiliations:** Department of Chemical Engineering, University of Chemistry and Technology Prague Technická 5, 166 28 Prague 6 Czech Republic viola.tokarova@vscht.cz

## Abstract

Microfluidic devices, allowing superior control over the spatial and temporal distribution of chemical substances and high process reproducibility, are nowadays essential in various research areas and industrial fields where the traditional “macroscopic” approach was no longer able to keep up with the increasing demands of high-end applications. In the present work, internal mixing of droplets formed by a flow-focusing X-junction at constant flow rates of both phases for three different channel heights (*i.e.* 20, 40 and 60 μm) was investigated and characterised. Both experimental methods and 3D CFD simulations were employed in order to resolve governing factors having an impact on internal mixing and homogenization time of model tracers inside of droplet reactors. Additionally, the influence of channel height on internal mixing was experimentally studied for continuous preparation of iron oxide nanoparticles by co-precipitation reaction. Since the initial nucleation phase is strongly affected by mixing and spatial distribution of all reactants, the final particle size and particle size distribution (PSD) can be used as direct indicators of mixing performance. It has been demonstrated that the smallest 20 μm channels provided narrower PSD and smaller particle mean size compared to higher channels.

## Abstract 2

Experimental and CFD numerical analysis of mixing efficiency in droplet-based microfluidics for various channel heights and its impact on the preparation of iron oxide nanoparticles.

## Graphical abstract

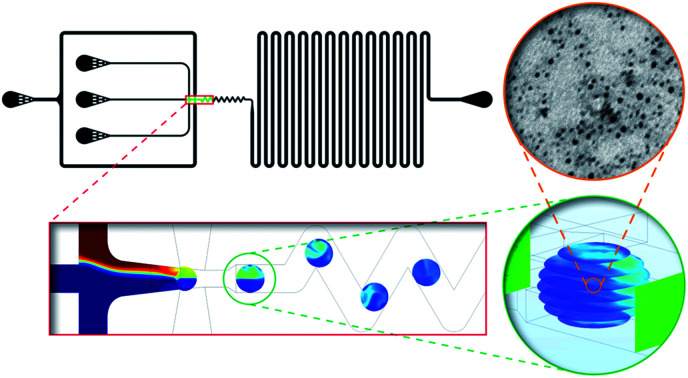

## 1. Introduction

Droplet-based microfluidics is an essential class of microfluidic devices developed for generation and handling of extremely tiny volumes with exceptional spatiotemporal precision. Droplet flow significantly eliminates the sample loss, cross-contamination, long diffusion times and Taylor dispersion effect often associated with a single-phase flow.^1,2^ With these unique advantages, droplet-based microfluidics is a preferred method of choice for a wide range of applications including chemical analysis and synthesis, as well as biochemical assays and high throughput screening demanding a thorough and rapid mixing of initially separated components.^3,4^

Monodisperse droplets can be generated in microfluidics actively using external source of energy (*e.g.* pressure pulses, vibrations, thermal field, electric field) or passively (pressure-driven flow).^5,6^ According to the chip layout and relative orientation of immiscible fluids, we can classify droplet generation geometries as (i) cross-flow,^7,8^ (ii) co-flow^9^ and (iii) flow-focusing.^10,11^ The cross-flow is formed by angled microchannels where dispersed and continuous phases come together (*e.g.* T-junction, Y-junction). In the co-flow geometry, the immiscible phases share the same flow direction in a set of coaxial microchannels. Flow-focusing geometry, employed in this work, is composed of three channels, one main channel and two symmetric side channels, forming axisymmetric X-junction where both immiscible fluids intersect each other. In this type of junction, one fluid (dispersed phase) is discretised at high frequency (Hz to kHz) into small volumes dispersed in the immiscible carrier fluid (continuous phase). The complex mechanism of droplet formation is based on competition among interfacial, inertia, viscous and gravitational forces. Contribution of the latter is due to small characteristic lengths usually negligible in microfluidics. For a given set of parameters, the interplay between acting forces governs the type of observed multi-phase flow pattern, *i.e.* co-flow, segmented flow, squeezing, dripping or jetting regime.^5^

Each droplet can be considered as a single reactor, physically and chemically isolated from the surroundings by the immiscible continuous phase. In some cases, a particular type of fluid is selected, allowing transport of necessary reagents across the droplet interface (*e.g.* CO_2_/O_2_ exchange essential for handling of living cells).^12^ Small droplet volumes and minimal reagent consumption, high interfacial area responsible for efficient heat and mass transfer, and isolation of droplet content from the surroundings are the most important for numerous high-end applications, *e.g.* single-cell handling, screening, and analysis,^4^ DNA encapsulation^13^ or nanoparticle synthesis and modification.^14,15^ In the case where two or more reacting streams form a droplet, efficient and very fast homogenisation have to be achieved. Internal mixing plays a crucial role in the applications where reaction mechanisms are very fast, typically in milliseconds, *i.e.* nucleation followed by particle growth in co-precipitation reactions.^16^ Fast homogenization in microfluidic devices is challenging, which is given by low values of Reynolds number (Re < 1) and laminar character of a flow, and mixing is only governed by molecular diffusion.^17^ Just as a droplet formation, enhancement of a droplet internal mixing can be achieved passively^18^ or actively^6^ using external energy, *e.g.* droplet-boundary oscillation under AC actuation,^19^ thermo-capillary mixing with micro-wave heater,^20^ droplet homogenization *via* electrostatic forces^21^ or magnetic actuation.^22^ Additional geometrical constraints can attain passive homogenization of a droplet content. The role of these structures is to introduce chaotic advection breaking axisymmetric recirculation profile inside of droplets, which results in enhancement of homogenisation without higher fabrication complexity of the device. Passive mixing can be achieved by non-rectangular channel cross-sections,^23^ and specific channel design (sinusoidal channels^24,25^ and baffle channels^26^) exploiting the formation of transversal vortices (Dean flows).^27^

Knowledge about hydrodynamics and mixing phenomena inside of droplets plays a vital role in design optimisation and prospective expansion of microfluidics in various high-end applications.^28,29^ Currently, the character of the fluid movement is mostly examined by microscale Particle Image Velocimetry (μPIV) using microscopic latex particles as a model tracer.^30^ For the experimental evaluation of mixing performance, a variety of microscopic techniques with the aid of flow visualisation by pH-sensitive, coloured and fluorescent dyes, have been employed.^14,31,32^ Two-dimensional projection of a 3D droplet into a plane has been addressed by Fluorescence-Lifetime Imaging (FLIM) providing three-dimensional information about a tracer distribution for both, quasi-steady flows^32^ and rapidly flowing droplets.^33^ However, a high purchasing cost significantly hinders the widespread application of this technique.

Numerical tools, on the other hand, allow addition insight into mixing phenomena with spatiotemporal resolution limited only by available computational resources. Most of the numerical studies about passive mixing in droplet-based microfluidics consider only lateral dimensions of channel geometry, and the influence of the channel height on droplet formation is omitted. Besides, mixing efficiency based on 2D CFD description of mass transport takes into consideration only an equatorial droplet cross-section which is not an accurate representation of a droplet volume and an internal recirculation flow pattern. Recently, 3D CFD analysis of internal mixing by convection has been demonstrated for slug-^34^ and droplet-flow generated by T-junctions.^35^ Despite the high popularity of droplet microfluidic technologies and increasing demand for fast and reliable mixing, three-dimensional numerical analysis of mixing performance of flow-focusing droplet generators, *i.e.* X-junctions, is still not fully explored.

In this work, we study and discuss the influence of the channel height on the droplet homogenization, taking into consideration the influence of horizontal channel dimension and 3D internal recirculation. The experimental results of mixing efficiency inside the droplet are substantiated by numerical simulations. Finally, the synthesis of iron oxide nanoparticles using droplet-based microfluidics, a model system of co-precipitation reaction highly sensitive to initial nucleation rate governed by initial mixing of precursors^36^ and their spatiotemporal distribution inside of droplet reactor, is provided. The influence of mixing performance for three channel heights (20, 40 and 60 μm) on the particle size and polydispersity index of prepared iron oxide nanoparticles is demonstrated. To the best of our knowledge, a similar study of X-junction droplet generator with various channel heights has not been conducted yet.

## 2. Material and methods

### 2.1. Materials

The following chemicals were used for the synthesis of iron oxide nanoparticles: iron(ii) chloride tetrahydrate (FeCl_2_·4H_2_O, Sigma Aldrich), iron(iii) chloride hexahydrate (FeCl_3_·6H_2_O, Merck), dextran 70 (*M*_w_ ∼ 70 kDa, Merck), ammonium hydroxide solution (NH_4_OH, 28.0–30.0 wt% NH_3_, Fluka), mineral oil (Sigma), methylene blue (P-LAB). Deionised water was filtered by Aqual 25 (conductivity ∼ 0.07 μS cm^−1^) and degassed before use. Polydimethylsiloxane (PDMS, Sylgard 184, Dow Corning) and silicone elastomer (Sylgard 184, Dow Corning) was used for the microfluidic chip fabrication.

Modular high-precision syringe pumps neMESYS (CETONI) were employed for accurate dosing and control of inlet flow rates for both dispersed and continuous phases. All liquids were filtered through a 0.25 μm filter to remove insoluble impurities before filling the syringes. All inlet/outlet capillaries (*d*_in_ = 0.32 mm) and fittings were made from Teflon (Adtech) to avert particle deposition and chip fouling. Olympus CKX41 inverted microscope with mounted Canon EOS 100D digital camera was used for visual observation of droplet flow and image acquisition. Fiji, a distribution of open-source ImageJ software, was used for all image post-processing, *i.e.* evaluation of droplet diameter and mixing performance.^37^ JEOL JEM-1010 (TEM – transmission electron microscope) at an acceleration voltage of 80 kV was used to observe the size and morphology of iron oxide nanoparticles. Micrographs were taken by SIS Megaview III digital camera (Soft Imaging Systems) and analysed by Fiji software. Comsol Multiphysics software was used for CFD simulation of the two-phase flow and reactant mixing. Interfacial surface tension *σ* and wetting angle *θ*_w_ were determined by optical tensiometer Attension Theta (Biolin Scientific). Dynamic viscosity of the fluid at room temperature was measured by rheometer Rheolab QC (Anton Paar GmbH). Surface Evolver – Fluid Interface Tool (SE-FIT) software^38^ was used for the numerical calculation of Laplace pressure for stagnant droplets by a gradient descent method.^39^

### 2.2. Chip design and fabrication

**Fig. 1:**
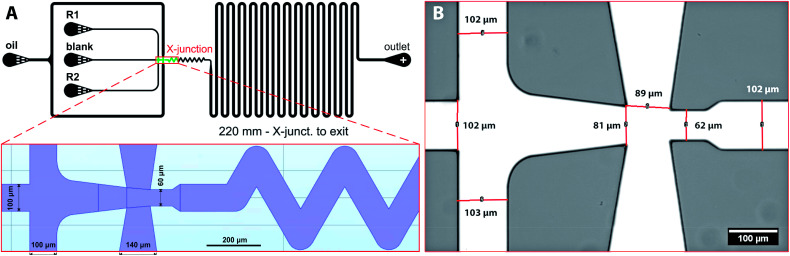
(A) The design of microfluidic chip for iron oxide nanoparticle synthesis with detail of flow-focusing junction; (B) a bright-field image of the flow-focusing X-junction of the assembled PDMS chip.

Planar chip design of the microfluidic chip was created in AutoCAD and converted in K-Layout software into GDSII format suitable for lithography. All dimensions and the detail of the flow-focusing junction are shown in Fig. 1 and SI(1B).† The final design has one outlet and four inlets, *i.e.* one for the continuous oil phase and three for dispersed phase – both reactants (R1, R2) and the blank solution. Droplets of the dispersed phase are formed at flow-focusing cross-junction (red rectangle in Fig. 1A) and are carried by continues oil phase towards the chip outlet *via* meandering and ageing channel with an overall length of 220 mm. Blank solution (pure water) is introduced to separate reactants before droplet formation and to reduce the possibility of a chip fouling caused by a gradual build-up of iron oxide precipitates in the cross-junction area.

Silicon masters with dykes of different heights (20, 40 and 60 μm) were prepared according to a previously published protocol.^14^ PDMS microfluidic chips were fabricated by a concentration gradient method where silicon master serves as a template, and the final chip is fabricated from two sandwiched layers of PDMS.^40^ This procedure ensures the same material properties of the chip governing wetting and droplet formation. Briefly, a degassed PDMS polymer mixture prepared by mixing the crosslinking agent and the silicone elastomer in mass ratio 1 : 10 was poured over the silicon wafer, degassed under vacuum to remove entrapped bubbles, and heat-treated at 75 °C for 22 min in the oven. Solidified PDMS mould was separated from the silicon master, and the connecting holes for inlet and outlet tubings were punched. The bottom part of the PDMS chip was fabricated from elastomer mixture poured into an empty Petri dish and heat-treated for 20 min at 75 °C. Due to a shorter heat treatment time, the upper surface of the PDMS layer remains sticky and adhesive. Both parts of the chip were gently assembled and placed in an oven at 75 °C to ensure proper bonding between the PDMS layers. Then, the inlet capillaries were connected to syringes (Hamilton) containing reagent solutions. The image of the whole microfluidic chip with connected capillaries is shown in ESI (Fig. SI(1)†). The detail of the cross-junction fabricated from PDMS is in Fig. 1B. A computer-controlled linear pump system (neMESYS) was used to precisely control the flow rates of the reagents and the continuous phase. The outlet was connected *via* 100 mm long capillary to a collection vial prefilled with pure water.

### 2.3. Microfluidic synthesis of iron oxide nanoparticles

Iron oxide nanoparticles were prepared by the reduction of iron cations using ammonium hydroxide solution as a reducing agent and dextran as a stabiliser according to previously published protocol with some modifications for continuous microfluidic preparation.^41^ The synthesis was performed at room temperature. All reactants were mixed in aqueous droplets surrounded by the continuous oil phase. In the first step, the dispersed phases labelled as R1 (Fig. 1A) was prepared by mixing 4.2 mg FeCl_3_·6H_2_O, 2.1 mg FeCl_2_·4H_2_O and 7.0 mg dextran 70 in 35 mL of demineralised water. The second dispersed phase (R2) was prepared by mixing 0.5 mL ammonium hydroxide solution with 0.5 mL of demineralised water. The third dispersed phase (“blank”) was demineralised water used as an inert phase wedged between R1 and R2, preventing premature reactant mixing and precipitation. The molar ratio of all reactants was kept the same for all preparations. The flow rates of each phase were controlled by the pump software GUI. The chip outlet was connected to a vial prefilled with water by 100 mm long capillary. Samples were collected after flow stabilisation period (up to 1 h) for 3 to 6 hours and centrifugated for 3 min at 13.4k rpm (MiniSpin Eppendorf) to separate oil from the aqueous phase. After removing the oil phase, samples were 3-times extracted with diethyl ether to remove remaining oil residues. Purified samples were sealed and stored at dark and cold conditions prior to the TEM analysis. Experiments, where flow oscillations, erratic flow or leakage were visually observed, were discarded from further analysis.

### 2.4. Evaluation of mixing efficiency

The reactant mixing inside droplets depends on several factors, *e.g.* fluid properties of both phases, flow rates, surface wettability. In this work, the influence of channel height on mixing behaviour was investigated. Methylene blue was used as a low molecular weight tracer (*M*_w_ = 319.9 g mol^−1^). Pre-filtered methylene blue solution was introduced into one dispersed phase (R1), and image series were captured using the camera mounted microscope for all three chip heights at fixed volumetric flow rates *Q* of both phases (continuous phase *Q*_c_ = 125 μL h^−1^, reactants *Q*_R1_ = *Q*_R2_ = 10 μL h^−1^, blank *Q*_B_ = 20 μL h^−1^, flow ratio between dispersed and continuous phase *Q*_r_ = *Q*_d_/*Q*_c_ = 0.32, where *Q*_c_ = *Q*_R1_ + *Q*_R2_ + *Q*_B_). Captured images were processed by Fiji software using following steps: (i) brightness and contrast adjustment of original RGB images; (ii) subtraction of image background to suppress light nonuniformity; (iii) splitting into colour channel layers – only red channel was used for further analysis; (iv) conversion into 8-bit grayscale; (v) histogram normalisation (pixel intensity with no dye set as pure white – pixel value 255 – and entering dye set as 0).

The region of interests (ROIs) was defined by the ROI Manager tool to manually define by ellipses of the same dimensions inscribed into droplets. Elliptical ROI was chosen over circular due to droplet deformation along the central line of the channel. These ROIs were equidistantly separated from dark droplet contours (caused by droplet curvature) by the margin of constant thickness in order to avoid false concentration overshoots. Example of image post-processing workflow is demonstrated in Result section 3.2 (Fig. 6A–C). Average pixel intensity and the standard deviation was evaluated in selected regions. Mixing intensity *m* was calculated for every droplet ROI based on mean pixel intensity *I*_avg_ and standard deviation of pixel intensity *σ* as:1
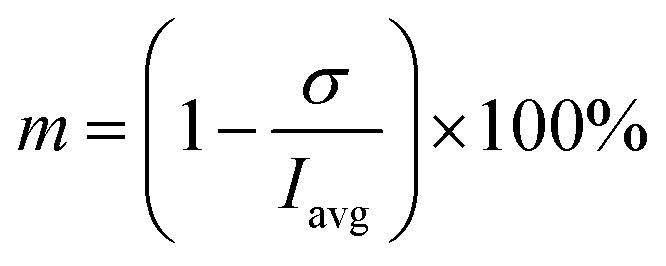


### 2.5. CFD study – two-phase flow

Comsol Multiphysics software (workstation Dell Precision Tower 7810, 2× Intel Xeon E5-2640, 64 GB DDR4 ECC + 500 GB of virtual M.2 SSD memory) was used for all CFD simulations, data post-processing and handling. Model of the flow-focusing junction with the adjacent meandering channel was imported into Comsol from the original CAD blueprint. Thanks to vertical symmetry, only one half of the chip was created as extrusion of planar geometry in *z*-axis direction (10, 20 and 30 μm for 20, 40 and 60 μm high channels). The final solution was obtained as a mirrored image of the solution merged with the original data set. This step is feasible thanks to the negligible influence of the gravity given by a small value of the Bond number (less than 1) defined as Bo = Δ*ρgh*^2^*σ*^−1^, where Δ*ρ* is the difference in densities of both phases and *h* is the hight of the channel. Experimentally obtained physical properties for dispersed phase (*ρ*_w_ = 987 kg m^−3^, *η*_w_ = 0.91 mPa s), continuous phase (*ρ*_oil_ = 854 kg m^−3^, *η*_oil_ = 26.06 mPa s), interfacial surface tension (*σ* = 47.5 mN m^−1^) and wetting angle of the dispersed fluid on flat PDMS substrate (*θ*_w_ = 20°) were used as input model parameters. The droplet generation was simulated by the laminar flow module (incompressible Newtonian fluids) coupled with the level-set method describing the evolution of the fluid interface. The flow of both phases was described by the equation of continuity (eqn (2)) and the Navier–Stokes equation for incompressible fluid without the contribution of gravity (eqn (3)). The level set function in a conservative form (eqn (4)) was used to preserve the overall mass of both phases. The governing equations are:

2 *ρ*∇ ·**u** = 0

3
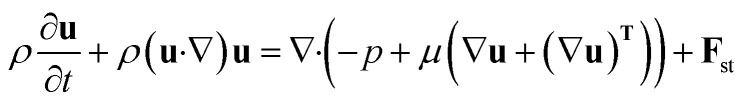

3.1 **F**_st_ = *σδκ***n** + *δ*∇_s_*σ*

3.2 *κ* = −∇·**n**

3.3 ∇_s_ = (**I** − **nn**^**T**^)∇

4
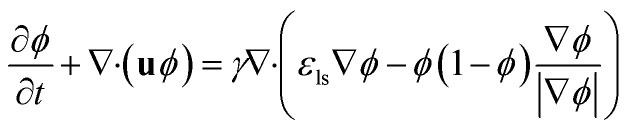
where vector **u** describes fluid velocity field, *p* stands for pressure, **F**_st_ is volumetric surface tension force acting on the interface between two fluids (N m^−3^), *σ* – interfacial surface tension (N m^−1^), *κ* – interface curvature (m^−1^), *δ* – Dirac delta function located at the interface, **n** – unit normal to the interface, ∇_s_ – surface gradient operator. In the eqn (4), two numerical stabilisation parameters (*ε*_ls_ and *γ*) responsible for the proper motion of fluid interface need to be manually adjusted. The *ε*_ls_ parameter controls the thickness of the interface and *γ* determines the amount of reinitialization. Based on Comsol documentation, the optimal *γ* and *ε*_ls_ values were set as maximum velocity magnitude (m s^−1^) and half of the maximal mesh size (m), respectively. Consequently, the mesh independence of the solution was verified solving the same problem scenario for decreasing mesh size and comparing results such as velocity at a given point, flow rate across control boundary, and droplet shape and droplet position at the same time. The mesh satisfying both result invariance and maximal RAM limit threshold was refined along the *z*-axis by factor 1.5 to obtain a sufficiently high resolution of the grid with 2.5, 5.2 and 7.9 × 10^6^ elements with a maximum mesh size of 3 μm. The time required to run one simulation was usually exceeding two weeks.

### 2.6. CFD study – mass transport and mixing

Mixing of the model tracer (inlet concentration – *c*_in_ = 1 mol m^−3^, diffusion coefficient – *D* = 10^−9^ m^2^ s^−1^) during the droplet formation and movement was numerically investigated for all channel heights. Previously calculated values of velocity and pressure field by the two-phase flow simulations were extended for mass transport equations describing convective and diffusion flux of the model tracer of concentration *c* in the aqueous phase expressed as:5
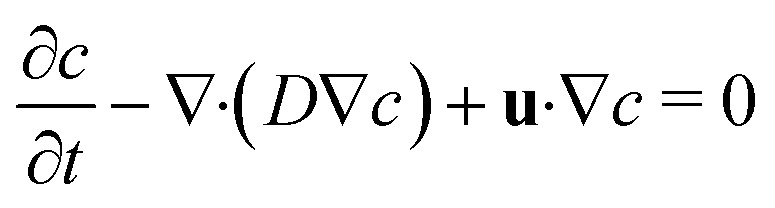


During the first series of trial simulations, we have encountered a problem with excessive diffusion of the tracer from droplets into the oil phase resulting in the gradual diminishing of the overall tracer amount inside of droplets. This effect was significantly reduced, introducing the level set-dependent velocity field and diffusion coefficient, both effectively preventing tracer flux across the droplet interface. The diffusion coefficient and velocity magnitude values were conditionally set to 0 m^2^ s^−1^ and 0 m s^−1^, respectively, at any point outside of aqueous phase (*Φ* < 0.5). For convection-dominated transport problems, Comsol Multiphysics automatically uses consistent stabilisation methods (streamline and crosswind diffusion) to prevent oscillations in the solution. However, these methods cause artificial diffusion and can create similar issues with excessive tracer dilution over time. On the other hand, simulations with disabled stabilisation often suffer from poor convergence and/or unphysical over- and undershoots (*e.g.* negative or very high local concentrations). This issue was solved using Do Carmo and Galeão consistent stabilisation, high-quality mesh and tight solver error tolerances at the cost of a high computational cost. Moreover, in order to account for the evolution of the concentration profile from the predetermined initial state (*t* = 0 s), the first three droplets were excluded from the analysis.

**Fig. 2:**
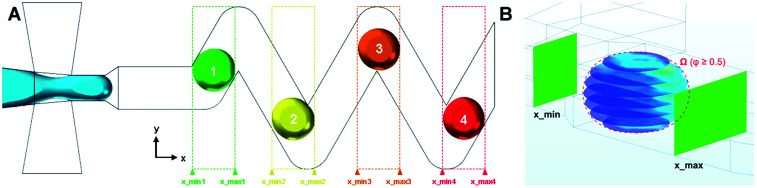
(A) Example of multiple spatial domains representing individual droplets in 60 μm chip, (B) parallel concentration profiles (in *xy* plane) defined in domain *Ω* (red dashed outline) where *Φ* ≥ 0.5 and *x* ∈ (*x*_min_; *x*_max_).

In order to study droplets as entities, continuous spatial domains *Ω* (*x*, *y*, *z*) corresponding to a droplet volume at a given time and space was determined. Domain *Ω* was only defined if both parameters, *Φ* ≥ 0.5 and *x* ∈ (*x*_min_; *x*_max_), were met. The first condition ensures that only the dispersed phase is considered, whereas *x*_min_ and *x*_max_ are *x* coordinates of the parallel boundaries fully confining one droplet at a time, as shown in Fig. 2.

The segmentation of the entire model into sub-domains allows to calculate the mean concentration of the tracer *c*_avg_, standard derivation of concentration *σ* and mixing segregation index *m* for every single droplet defined as:6
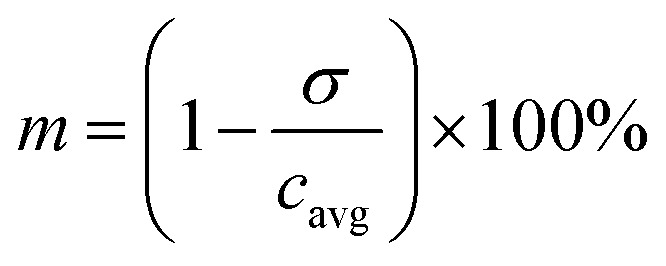
6.1
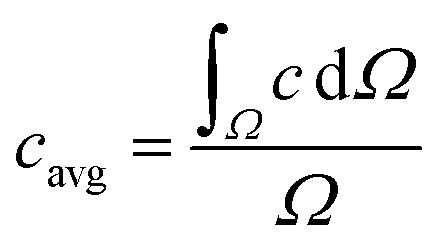
6.2
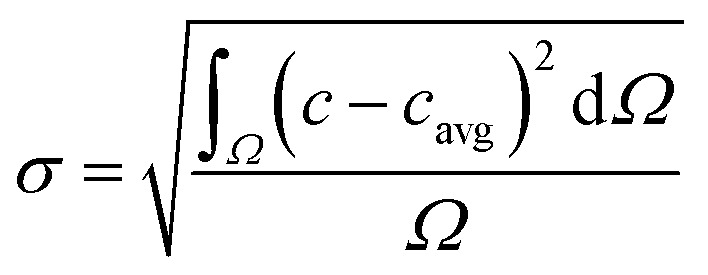


## 3. Results

### 3.1. Droplet generation – CFD *vs.* experiments

Numerical simulation of the two-phase flow was used for identification of suitable flowrates of all inlet streams and their respective ratios ensuring stable droplet generation at an adequate frequency preventing coalescence of adjacent droplets travelling one-by-one through the ageing channel. Based on numerical results, the flow rates of the continuous oil phase, reactants (R1, R2) and blank were set as 125 μL h^−1^, 10 μL h^−1^ and 20 μL h^−1^ for all channel heights, respectively. These values were kept constant for all experiments and numerical simulations in order to study the influence of channel height on the mixing performance only.

**Fig. 3:**
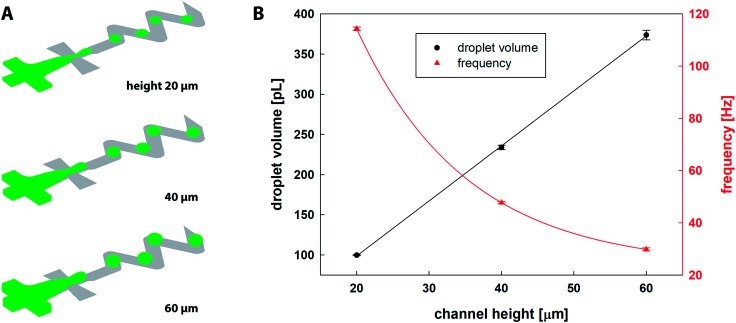
(A) 3D CFD simulations of droplet generation in a microfluidic chip for three different channel heights (20, 40 and 60 μm); (B) droplet volume [pL] and droplet generation frequency [Hz] as a function of channel height (averaged numerical results of five individual droplets).

The CFD results of the droplet generation are shown in Fig. 3A. Fiji software was used for calculation of droplet diameter *d* = (4*A*π^−1^)^0.5^ based on the surface area *A* of a droplet equatorial cross-section. Table 1 summarises numerical and experimental results and Fig. 3B shows the relation between the channel height and the droplet diameter/volume/frequency. It was observed that for the fixed inlet conditions, the microfluidic chip with higher channels produces larger droplets, and 2× and 3× higher channels resulted in 14% and 21% increase in droplet diameter, respectively. The volume of dispersed droplets was linearly proportional to a channel height. Frequency of droplet generation observed for 20 μm channels (114.3 ± 0.7 Hz) was reduced by 58% and 74% for 40 and 60 μm channel height, respectively. The separation distance between two consecutive droplets measured by Fiji software along the central line of the channel was larger with channel height, *i.e.* 339, 350 and 365 μm for 20, 40 and 60 μm, respectively.

**Table 1:** Comparison of droplet diameter based on experimental and CFD results, droplet volume and frequency of droplet generation

Height [μm]	*d*_exp_ [μm]	*d*_sim_ [μm]	Δ*d* [%]	*V*_sim_ [pL]	*f*_sim_ [Hz]	Ca [—]
20	84.8 ± 1.7	81.8 ± 0.1	3.5	99.7 ± 0.5	114.3 ± 0.7	12.57 × 10^−3^
40	97.2 ± 0.3	93.4 ± 0.3	2.0	233.8 ± 2.8	47.7 ± 0.4	6.29 × 10^−3^
60	103.6 ± 5.7	99.2 ± 0.8	4.3	373.7 ± 6.1	29.8 ± 0.6	4.19 × 10^−3^

For experimental results, the microscopy images of droplets were evaluated using Fiji software. The minimum of 30 droplets from three independent experiments of each channel height were evaluated and compared. Experimental results are in agreement with simulation results in terms of droplet size and spacing. The droplet sizes based on CFD results were slightly underestimated (Δ*d* = 2.0 to 4.3%), as shown in Table 1. The last column shows the calculated capillary number (Ca) defined as Ca = *η*_oil_*uσ*^−1^, where *u* is a characteristic velocity of the fluid in the outlet channel (100 μm width), *η*_oil_ is the dynamic viscosity of the continuous phase, and *σ* is interfacial surface tension.

**Fig. 4:**
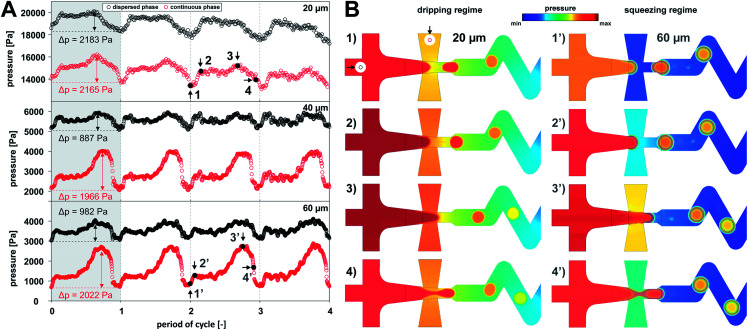
(A) Evolution of pressure for continuous (red) and dispersed phases (black) during four droplet formation cycles; (B) pressure profiles for dripping (1–4) and squeezing regime (1′–4′) for 20 and 60 μm high channels, respectively. Pressure probes were *z*-positioned in the middle of the model near the entrance of both phases as indicated by black arrows in inset image (1).

Numerical simulations characterised the flow regimes for all studied channel heights. In terms of a droplet generation in X-junction, one can distinguish three main flow regimes, (i) dripping, (ii) squeezing and (iii) jetting.^14^ Droplets formed in the jetting regime are detached at the end of the elongated thread of the dispersed phase outside of the focusing junction. The jetting regime was not observed for given combinations of flow rates and channel heights. Dripping regime can be distinguished from squeezing regime by the fact the continuous phase is not fully separated by the dispersed phase at any moment. In the case of squeezing regime, the dispersed phase obscures outlet orifice completely, which results in a gradual increase of upstream pressure in the continuous phase. Relationship between the pressure and the droplet formation period is shown in Fig. 4. The droplet formation of the dripping regime, observed only for a 20 μm high channel, can be divided into following stages: (a) droplet pinch off and recoil – the minimal pressure of both phases; (b) gradual interface growth; (c) partial blocking of outlet channel – the highest pressure in the continuous phase (Δ*p* = 2165 Pa); (d) necking followed by the droplet detachment (stage a). On the other hand, squeezing regime was characteristic for 40 and 60 μm channel heights where dispersed fluid fully occupied outlet channel. This regime can be described by the following stages: (a′) droplet detachment – the lowest pressure for both phases; (b′) interface bulging; (c′) channel clogging – gradual increase of pressure in continuous phase to the maximum value (Δ*p* was 1966 and 2022 Pa for 40 and 60 μm channels, respectively); and (d′) necking followed by droplet formation.

**Fig. 5:**
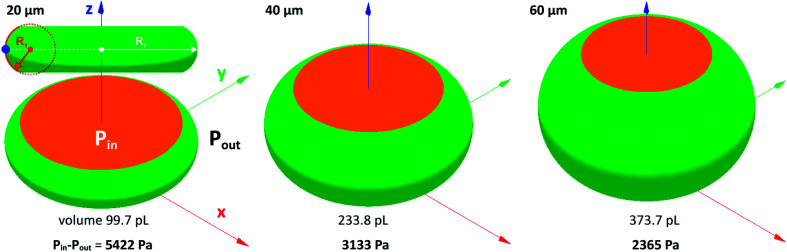
Surface Evolver simulation of droplets squeezed between two parallel planes with corresponding values of Laplace pressure for 20, 40 and 60 μm high channels. Orange and green colours represent droplet contact with fixed horizontal planes and mobile droplet interface, respectively.

The pressure difference across the interface of relaxed droplets corresponds primarily to the Laplace pressure (Δ*p* = *σ*(1/*R*_1_ + 1/*R*_2_), where *R*_1_ and *R*_2_ are principal radii of curvature at a given point on the free droplet interface). Numerical calculations of pressure across the interface of steady droplets were obtained using Surface Evolver software. Droplet volume (Table 1) was confined between the two parallel planes separated by the distance corresponding to a channel height. Model inlet parameters (*i.e.* liquid density, interfacial surface tension and contact angle) were the same as for Comsol simulations described in Section 2.3. The droplets with minimal surface energy are shown in Fig. 5 with calculated values of Laplace pressure: 5422, 3133 and 2365 Pa for 20, 40 and 60 μm high channels, respectively. The increasing pressure for lower channels is caused by a smaller radius of curvature *R*_1,_ as illustrated in Fig. 5 by red dashed circle.

### 3.2. Experimental evaluation of mixing

**Fig. 6:**
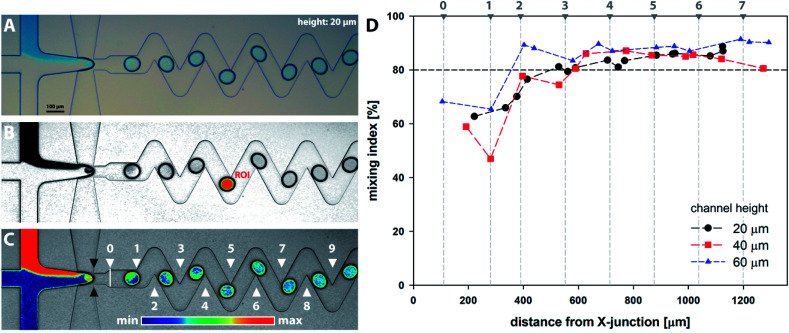
Experimental evaluation of mixing intensity: (A) original bright field microscopy image; (B) adjusted and normalised grayscale image with highlighted ROI region (red) separated from droplet outline (black) by a constant margin (yellow); (C) image overlay with the description of bends (white △) and cross-junction (black ▲). Images (A–C) represent the 20 μm high chip; (D) mixing intensity based on the horizontal distance of droplet centre and cross-junction for 20, 40 and 60 μm high channels.

Series of microscopy images with a model dye introduced as solution R1 were acquired and analysed as described in Section 2.4 and are demonstrated in Fig. 6A–C. Results for all channel heights based on horizontal distance from the cross-junction are summarised in Fig. 6D. The distance is calculated as the horizontal span between the ellipse centre and the junction (showed by black marks in Fig. 6C). From the results, it can be observed that the homogenization (mixing index *m* ≥ 80%) occurs for all channel heights after the 3rd bend mark (showed by white marks in Fig. 6C). It should be noted the 2D microscopy images provide no information about the tracer distribution alongside the channel height. Furthermore, additional factors can significantly affect the final data interpretation, *e.g.* camera resolution, quality of optical elements, colour artefacts, motion blur associated with a droplet movement and oscillations, nonuniformity of light distribution and validity of Lambert–Beer law across the concentration range.

### 3.3. CFD analysis of mixing

**Fig. 7:**
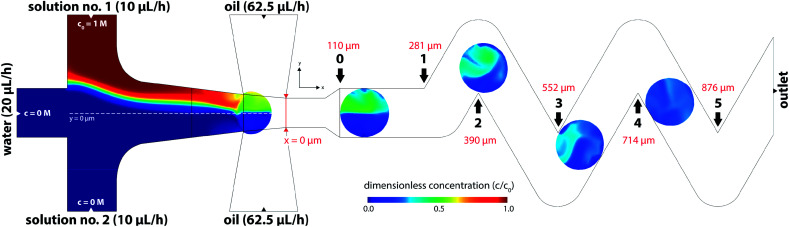
Distribution of a model tracer in the equatorial plane (*z* = 30 μm) of the 60 μm height microfluidic chip. Black arrows show *x*-coordinates of bend positions. Note: model tracer is introduced only in solution no. 1 (R1).

CFD numerical tools offered significant advances over most of the experimental methods and were used for evaluation of the tracer distribution and mixing in the volume. Using this approach, a spatial resolution of the tracer distribution and homogenization is otherwise experimentally unreachable. Fig. 7 shows a planar concentration profile of 60 μm high channel with a dematerialised continuous phase for better visibility and description of the inlet conditions. The tracer (concentration 1 M) is present as solution no. 1 (R1) at a flow rate of 10 μL h^−1^.

**Fig. 8:**
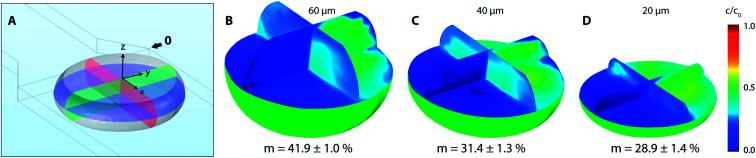
(A) Droplet tangent to reference point 0 with highlighted perpendicular planes of interest (*xy*, *xz* and *yz*) in 20 μm high channel; (B–D) mixing index and 3D distribution of tracer inside of droplets for 60, 40 and 20 μm high channels at the same position.

Based on experimental observations and numerical results, inlet streams of the dispersed phase are effectively separated by the blank phase ahead of the cross-junction. The first significant mixing of reacting streams (R1 and R2) occurs near the liquid–liquid interface preceding droplet formation by the mechanism known as the twirling effect.^28^ Three-dimensional tracer distributions and mixing indices for droplets exiting the outlet nozzle (position 0 in Fig. 7) are shown in Fig. 8. Interestingly, the calculated mixing intensity increased with the channel height, even though the droplet volume and diffusion lengths are both larger. This can be explained by tracer distribution and increased mixing at the junction since the lower frequency of the droplet generation for higher channels leads to a longer duration of the premixing period expressed as *T* = *f*^−1^, where *f* is the frequency of the droplet generation. Therefore, the initial spatial distribution of the tracer at the time of a droplet formation is significantly affected by the duration of premixing period *T*, *i.e.* 9, 21 and 34 ms for channel height 20, 40 and 60 μm, respectively.

**Fig. 9:**
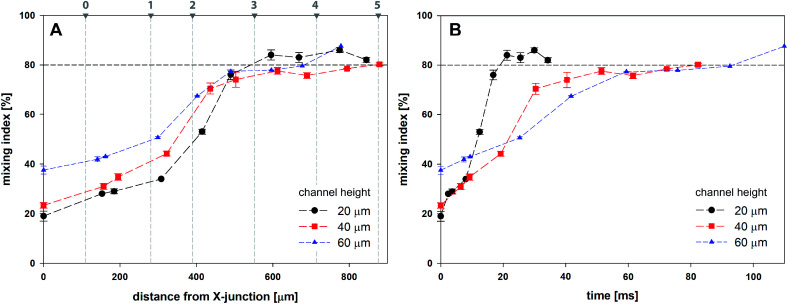
Mixing index for droplets as a function of channel height based on (A) the distance from X-junction; (B) time.

Fig. 9A shows the evolution of mixing index *m* for 20, 40 and 60 μm high channels based on the horizontal distance of the droplet centre from the cross-junction. Every data point represents the mean value of five consecutive droplets captured at the same position, which was achieved manually by superposition of the droplet outlines.

Initial droplet mixing evaluated at the time of droplet formation (before Position 0 in Fig. 7) was significantly higher for 60 μm channel compared to 40 and 20 μm channels, *i.e.* 37.6% *vs.* 23.3% and 19.0%, respectively. The higher *m* values caused the prolonged duration of premixing period, which was nearly 4-times longer for 60 μm than that for 20 μm high channels. Differences in mixing intensities were gradually reduced with increasing distance from the junction. Mixing intensities were comparable for all heights at the 3rd bend mark, although only 20 μm channel reached the threshold of 80%. Mixing index for 40 and 60 μm channel heights exceeded 80% after the 5th and the 4th bend mark, respectively. In comparison, 20 μm channel height provided the quickest homogenization, followed by 40 and 60 μm channel heights. The initial highest mixing index for 60 μm channels was outperformed by 20 and 40 μm height channels in 10 and 21 ms, respectively. Time to achieve *m* > 80% was evaluated to be 19 ms, 82 ms and 93 ms for 20, 40 and 60 μm channel height. The information about the time (droplet age) and mixing index showed in Fig. 9B is particularly important in the area of very fast precipitation reactions where nucleation and seed formation occurs almost instantly upon reactant mixing.

### 3.4. Preparation of iron oxide nanoparticles

Iron oxide nanoparticles were prepared according to the procedure described in Section 2.3 using the microfluidic chip at room temperature. Nanoparticles formed in individual droplets were collected in vials prefilled with water which dilutes the sample and instantly quench the reaction. Collected iron oxide nanoparticles suspended in an aqueous phase were protected from oxidation by the continuous oil phase also presented in the collection vial. Estimated residence time for one droplet travelling *via* meandering, ageing channel and 100 mm long outlet capillary into a collecting vial was approx. three minutes – 185, 195 and 204 s for 20, 40 and 60 μm high channels, respectively. The residence time of the droplet, defined as the time interval between droplet formation and collection, can be considered as the overall duration of the co-precipitation reaction.

**Fig. 10:**
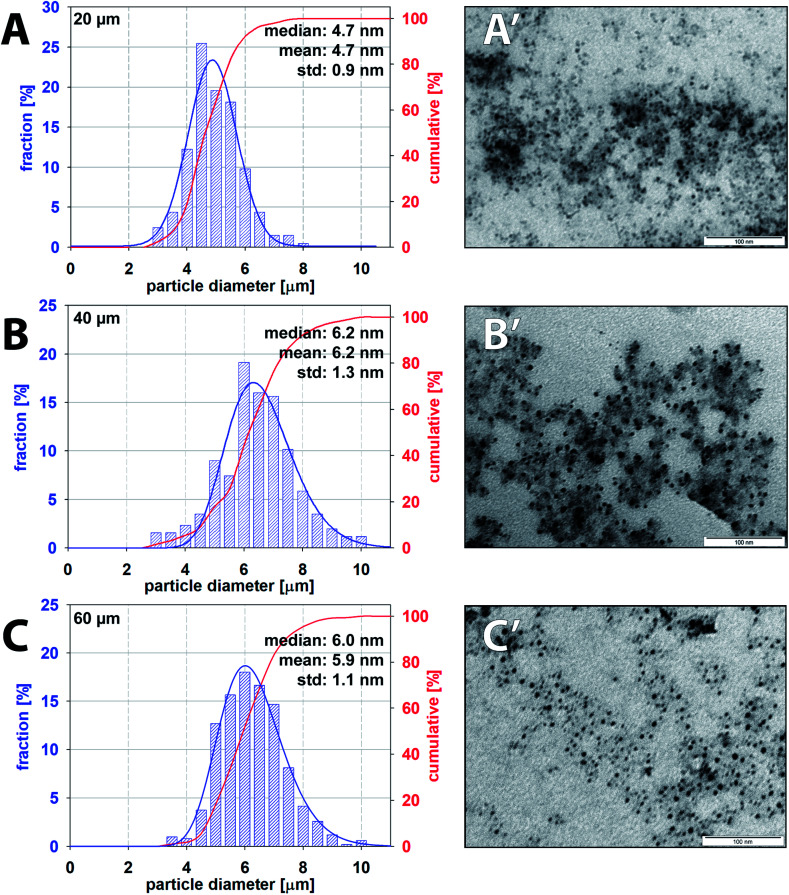
Particle size distribution and corresponding TEM images of iron oxide nanoparticles produced by 20 μm (A and A′), 40 μm (B and B′) and 60 μm (C and C′) high microfluidic channels.

TEM was employed as the preferred technique for direct visualisation of the sample and Fiji software for nanoparticle characterisation. TEM images of samples collected from three independent experiments for each channel height were analysed with the total number of measured particles exceeding 500. Averaged particle size distribution (PSD) of iron oxide nanoparticles for all channel heights with respective values of mean and median size are summarised in Fig. 10. Additional TEM images can be found in the ESI (Fig. SI(2)†). The mean size of the observed particle was evaluated to be 4.7 ± 0.9 nm, 6.2 ± 1.3 nm and 5.9 ± 1.1 nm for 20, 40 and 60 μm high channels. Nanoparticles produced by 20 μm high channel showed the narrower PSD in comparison to 40 and 60 μm channels. PSD for higher channels were considerably broader, and this observation can be explained by a slower internal homogenization (*m* > 80%) of reactants for 40 and 60 μm channels (82 ms and 93 ms, respectively) compared to 20 μm high channel (19 ms) as discussed in Section 3.3. TEM images revealed a spherical shape of nanoparticles without the presence of larger agglomerates or overgrown crystals in all cases. The polydispersity indices defined as PDI = (the standard deviation/mean particle diameter)^2^ for increasing channel heights were 0.037, 0.044 and 0.035, respectively. Importantly, the obtained nanoparticles with monomodal distribution (PDI < 0.1) were prepared at room temperature in contrast to a standard batch preparation^41^ or droplet capillary reactors^42^ operating at elevated temperature.

## 4. Conclusion

The presented work describes the influence of channel height (*i.e.* 20, 40 and 60 μm) on mixing performance within the droplets produced by microfluidic flow-focusing X-junction using experimental and numerical approach. The mixing indices were evaluated from the 2D experimental setup and compared to the 3D CFD model of the tracer distribution, and both results were in agreement. The fastest homogenization time (*m* > 80%) of reactants inside of a droplet was by far achieved for the smallest channel height, *i.e.* 20 μm. This is an important finding with regard to sensitive and very fast chemical reactions, such as co-precipitation reactions. The model reaction system of iron oxide nanoparticles prepared by the co-precipitation method using droplet flow microfluidics was demonstrated. Moreover, the synthesis was performed at room temperature in contrast to the elevated temperature usually used for batch-wise synthesis. From the obtained nanoparticle samples, it was found the relationship between time-dependent droplet homogenization and the final iron oxide nanoparticle size distribution. The 20 μm channel height provided the smallest nanoparticles compared to 40 and 60 μm channel heights. On the other hand, the highest channel, *i.e.* 60 μm, provides more uniform nanoparticles compared to 40 μm channel which can be attributed to a longer premixing period during the interface bulging, resulting in higher initial mixing prior droplet formation. The higher channels are favourable for applications where adsorption of reaction products on walls of the microfluidic chip is unfavourable, and the contact interface between walls and droplet must be reduced to a minimum, *e.g.* preparation of silica particles in glass/PDMS microfluidics. Detailed knowledge about the time-dependent internal mixing based on the height of a microfluidic chip can help to predict properties of the final product. The optimisation of a microfluidic design can be tailored for applications where reproducible, fast mixing and well-defined reaction time are needed.

## Conflicts of interest

There are no conflicts to declare.

## Supplementary Material

RA-010-D0RA02470H-s001
