# Supplementary material for: Influence of channel height on mixing efficiency and synthesis of iron oxide nanoparticles using droplet-based microfluidics
Source: RSC Adv. 2020 Apr 17;10(26):15179–89. doi: 10.1039/d0ra02470h (PMC9052325; doi:10.1039/d0ra02470h)
Supplement: RA-010-D0RA02470H-s001 [file RA-010-D0RA02470H-s001.pdf]

Electronic Supplementary Material (ESI) for RSC Advances.
This journal is © The Royal Society of Chemistry 2020

Supporting Information

Influence of channel height on mixing efficiency and synthesis of iron oxide nanoparticles using droplet-based microfluidics

O. Kašpar, A. H. Koyuncu, A. Hubatová-Vacková, M. Balouch, V. Tokárová*

Department of Chemical Engineering, University of Chemistry and Technology Prague, Technická 5, 166 28, Prague 6, Czech Republic, *viola.tokarova@vscht.cz

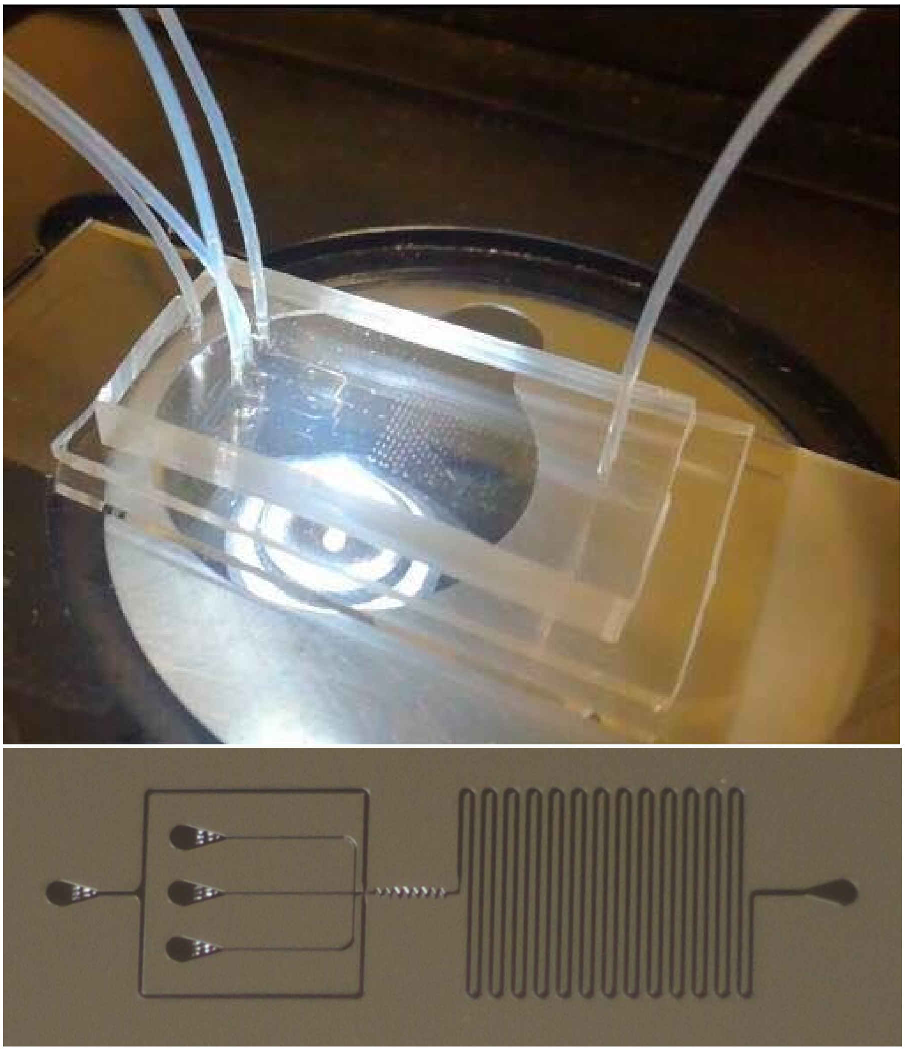

A)

B)

Fig. SI 1.: A) Image of the assembled microfluidic chip connected to syringe pumps (four inlets) and one outlet channel for collecting the sample. The droplets are visible in the ageing channel, B) silicon wafer used for replication of the chip channel structure in PDMS

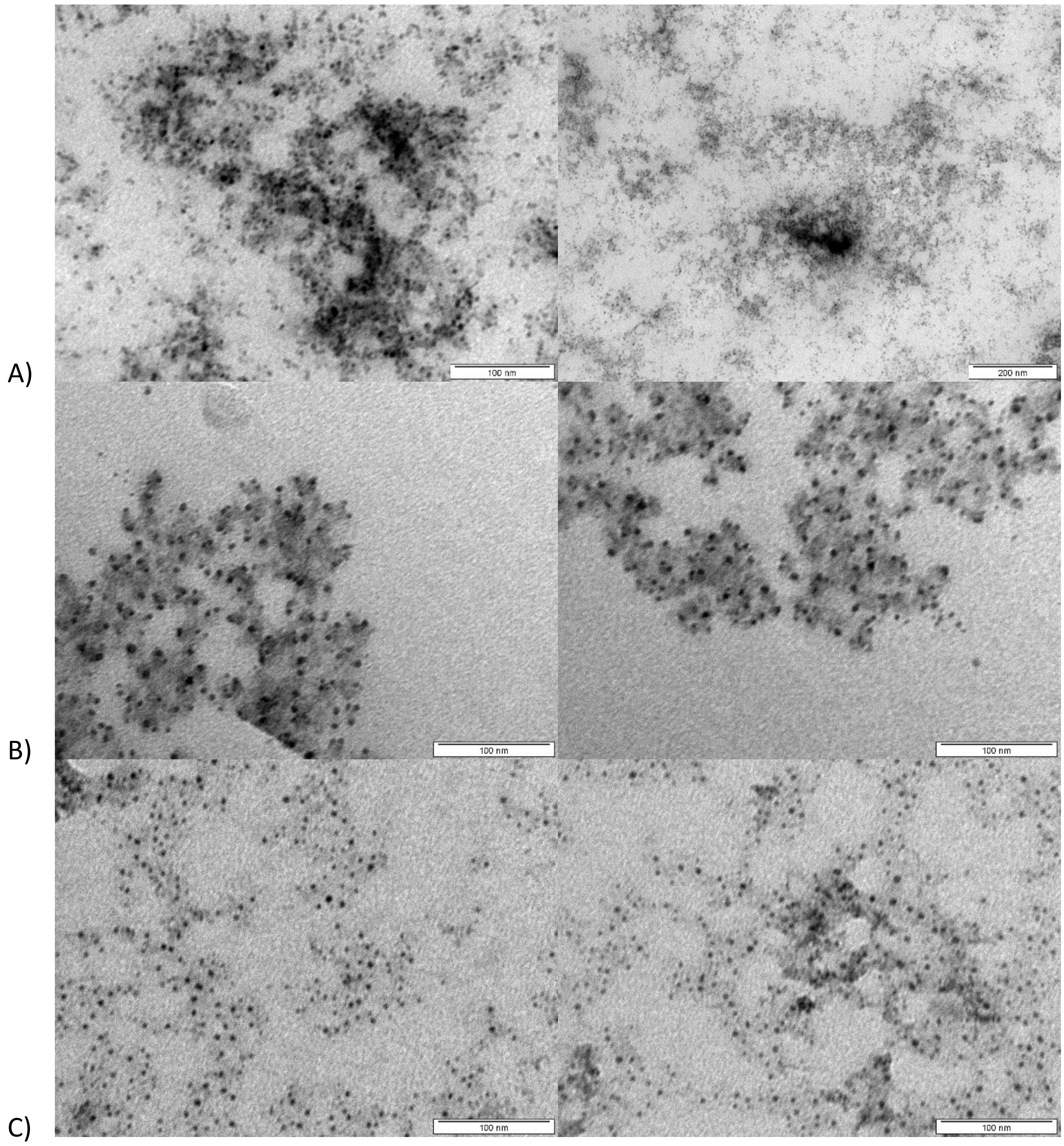

Fig. SI 2.: TEM images of iron oxide nanoparticles synthethized in microfluidic chip of deffirent channel height: A) 20 µm, B) 40 µm, C) 60 µm
